# Association of Metabolic Dysfunction-Associated Steatotic Liver Disease/Non-alcoholic Fatty Liver Disease With Type 2 Diabetes Mellitus: A Case-Control Study in a Tertiary Care Hospital in Pakistan

**DOI:** 10.7759/cureus.47240

**Published:** 2023-10-17

**Authors:** Faizan Zahoor, Najam-us-Sehar Saeed, Salman Javed, Hafiz Zeeshan Sadiq, Fasih Mand Khan, Miqdad Haider, Muhammad Nabeel Shafqat, Arman Maqbool, Auj Chaudhry

**Affiliations:** 1 Department of Medicine, Allied Teaching Hospital Gujranwala, Gujranwala, PAK; 2 Department of Gastroenterology and Hepatology, Allied Teaching Hospital Gujranwala, Gujranwala, PAK; 3 Department of Gastroenterology and Hepatology, Services Hospital Lahore, Lahore, PAK; 4 Department of Medicine, Aziz Bhatti Shaheed Teaching Hospital, Gujrat, PAK; 5 Department of Medicine, Fatima Memorial Hospital College of Medicine and Dentistry (FMHCM&D), Lahore, PAK; 6 Department of Family Medicine, Health Education England, Birmingham, GBR; 7 Department of Gastroenterology and Hepatology, PARSA Trust Clinic, Gujranwala, PAK

**Keywords:** nonalcoholic fatty liver disease (nafld), bmi, steatosis, type ii diabetes mellitus, fatty liver, metabolic dysfunction-associated steatotic liver disease

## Abstract

Background

Type 2 diabetes mellitus (T2DM) is a chronic metabolic disorder characterized by high blood sugar levels, insulin resistance, and relative insulin deficiency. Metabolic dysfunction-associated steatotic liver disease (MASLD) is the term used to describe fatty liver (steatosis) in individuals without a history of significant alcohol intake. MASLD is progressively known as the leading cause of chronic liver disease. Dietary factors, a significant risk factor for developing T2DM and cardiovascular disease, also contribute to MASLD development. The risk of developing MASLD increases with age, particularly in patients with diabetes mellitus. This risk is notably elevated among South Asians due to their higher incidence of insulin resistance and metabolic syndrome. Importantly, MASLD is acknowledged as a component of the metabolic syndrome, with insulin resistance playing a central role in its development.

Objective

To determine the association between MASLD and T2DM in patients presenting at a tertiary care hospital in Pakistan.

Methodology

This case-control study was conducted for one year in a tertiary care hospital in Gujranwala, Pakistan. A total of 380 patients were enrolled through convenient sampling and were analyzed according to two groups: those with diabetes (case) and those without diabetes (control). All participants were assessed for serum aspartate transaminase (AST) and alanine transaminase (ALT) levels and underwent abdominal ultrasound to determine hepatic fibrosis. A diagnosis of MASLD was made only in the presence of hepatic steatosis with AST and ALT values of more than 40 IU. The odds ratio (OR) was calculated, and stratified analysis was conducted according to gender, age, and BMI. A p-value of ≤0.05 was considered statistically significant.

Results

In our study, 55.53% of patients were male, while 44.47% were female. The average BMI (±SD) of the patients was 23.66±3.08 kg/m2. Among the cases group, the MASLD was noted in 91 (47.9%) patients, while among the controls group, the MASLD was noted in 64 (33.7%) patients with a statistically significant OR of 1.810 (1.19-2.74).

Conclusion

In conclusion, MASLD is significantly associated with T2DM, regardless of gender and BMI of patients. We recommend screening T2DM patients for the presence of MASLD at regular intervals to prevent hazardous consequences of MASLD in adult populations, particularly those with features of metabolic syndrome. Further larger-scale studies investigating the impact of T2DM on MASLD are required to reduce morbidity and decrease disease burden, especially in prevalent areas.

## Introduction

Metabolic dysfunction-associated steatotic liver disease (MASLD) is a complex and multifaceted disease that is increasingly recognized as a significant cause of chronic liver disease and encompasses a spectrum of liver pathologies. It starts with steatosis, the accumulation of fat in the liver, leading to metabolic dysfunction-associated steatohepatitis (MASH), a more severe inflammatory disorder, and can ultimately progress to cirrhosis and hepatocellular carcinoma [1-3]. MASLD is a widespread chronic condition of which diabetic fatty liver accounts for a significant proportion, with ultrasonography revealing fat accumulation in the liver in 50-75% of individuals with type 2 diabetes mellitus (T2DM) [4, 5]. The increasing global prevalence of MASLD is attributed to the rising epidemic of T2DM, hypertension, obesity, and hyperlipidemia [6-9]. Although the increasing prevalence of MASLD is frequently attributed to the epidemic of obesity and is often oversimplified as the “hepatic manifestation of the metabolic syndrome,” it is a much more complex disease process that may also be observed in non-obese individuals and patients without clinical manifestations of the metabolic syndrome [5, 10-12]. Because MASLD is a complex disease with both metabolic and liver-specific complications, its treatment approach is unique among medical disorders [7, 9, 13-15]. T2DM contributes significantly to the development of MASH, the more severe form of MASLD, and increases the risk of cirrhosis and hepatocellular carcinoma [12-14, 16]. The presence of hyperinsulinemia or insulin resistance, common features of the metabolic syndrome, suggests that MASLD could represent the liver component of this condition [15]. South Asians, in particular, have a high prevalence of both insulin resistance and metabolic syndrome, rendering them more susceptible to MASLD [17]. Currently, MASLD is acknowledged as a component of the metabolic syndrome, with insulin resistance playing a crucial pathogenic role [17, 18].

Patients with T2DM, in particular, exhibit histologically more severe MASLD. However, MASH and advanced fibrosis can still occur in a considerable proportion of MASLD patients without T2DM. The lower utility of the MASLD fibrosis score in MASLD patients without T2DM emphasizes the heterogeneous nature of the MASLD phenotype [13, 18]. MASLD and T2DM are common conditions that regularly co-exist and can act synergistically to drive adverse outcomes. For instance, their coexistence can increase the likelihood of developing complications associated with T2DM (including both macro and microvascular complications) and exacerbate the severity of MASLD, including cirrhosis, hepatocellular carcinoma, and death [19]. The prevalence of MASLD was substantially higher in individuals with T2DM (28.3%) than in those without T2DM (17.5%), and the difference was statistically significant (p<0.001) [20]. The objective of this study was to determine the association of MASLD with T2DM in patients presenting at Allied Teaching Hospital Gujranwala, Pakistan. Through literature, it has been noticed that there is a significant association of MASLD with T2DM. Furthermore, no local studies have been discovered that might assist us in confirming whether MASLD is strongly linked with T2DM. As a result of the absence of data, there is a need to screen T2DM patients for the existence of MASLD. So that we can establish a strategy to screen the adults while they are gaining weight to determine the risk of MASLD and the risk of getting T2DM.

## Materials and methods

Study sample and design

A case-control study looking at a period of one year was carried out at Allied Teaching Hospital Gujranwala, Pakistan, starting from January 2022 up to January 2023. The study was approved by the Institutional Review Board of Gujranwala Medical College/DHQ Teaching Hospital, Gujranwala, with the following IRB approval letter number 212/23. The study's sample size is 380, estimated using 80% study power, 5% significance level, assumed odds ratio of 2, and the predicted proportion of MASLD, i.e., 28.3% with T2DM and 17.5% without T2DM [20]. The sample was collected through a non-probability, consecutive sampling technique.

Inclusion criteria

The inclusion criteria for the study were as follows: Participants had to be between the ages of 35 and 80 years and could be of any gender. The case group comprised patients diagnosed with T2DM. In contrast, the control group included participants without T2DM, who were normal, healthy relatives of the patients with diabetes. 

Exclusion criteria

Participants were excluded from the study if they had any systemic issues such as renal disease, evident by creatinine levels exceeding 1.2 gm/dl, or if they were already administering statins for lipid management, as documented in their medical records. Additionally, anyone with a past incidence of hepatitis B (HBV) or hepatitis C (HCV) infection was not considered. The exclusion criteria also applied to alcoholics (on history) and individuals with a smoking history of more than five pack-years.

Data collection

After obtaining informed consent, 380 participants (190 in each group) who fulfilled the inclusion and exclusion criteria and presented to the hospital's outpatient service were enrolled. The following demographic data was gathered: name, age, gender, BMI, and, in some cases, the length of T2DM. Patients were divided into two groups, i.e., cases with T2DM and controls without T2DM. Blood samples were obtained in a 3 cc syringe and sent to the hospital's laboratory to assess aspartate transaminase (AST) and alanine transaminase (ALT) levels. To evaluate hepatic fibrosis, abdominal ultrasonography was performed on all participants. Patients with elevated aminotransferases (ALT and/or AST levels > 40 IU) and hepatic steatosis were diagnosed with MASLD. All this information was recorded on a proforma.

Data analysis

The collected information was entered into SPSS version 21.0 and analyzed through it. Quantitative variables such as age, BMI, and duration of T2DM in cases were calculated as mean ± SD. Categorical variables such as gender and MASLD were expressed as frequency and percentage. The OR was calculated to measure the association between MASLD and T2DM. Data were stratified by age, gender, and BMI. After stratification, the OR was recalculated to measure the association between MASLD and T2DM for each stratum separately.

## Results

The general demographic characteristics of the normally distributed study population are shown in Table 1. Study participants' mean age (±SD) was 55.14 (±55.14%). The average BMI was 23.66 (±3.08). Out of the total 380 participants, 211 (55.53%) patients were male while 169 (44.47%) patients were females, with a male-to-female ratio of 1.2:1. In the cases group, the number of male and female patients was 103 (54.2%) and 87 (45.80%). In contrast, in the control group, it was 108 (56.80%) and 82 (43.20%), respectively. No significant difference was noted in the distribution of gender, age, and BMI among case and control groups (Table 1).

**Table 1:** Demographic characteristics of study participants according to baseline type 2 diabetes mellitus status.

		Study Groups			Total
		Case	Control	P-value	
Age (years)	n	190	190		380
	Mean (±SD)	55.54 (±11.22)	54.73 (±11.49)	0.45	55.14 (±11.35)
BMI (Kg/m^2^)	Mean (±SD)	23.62 (±3.19)	23.72 (±2.98)	0.75	23.66 (±3.08)
Gender	Male (%)	103 (54.20%)	108 (56.80%)	0.68	211 (55.53 %)
	Female (%)	87 (45.80%)	82 (43.20%)		169 (44.47 %)

According to the study results, a total of 155 (40.79%) patients were diagnosed with MASLD, whereas 255 (59.21%) did not have MASLD. The frequency distribution of MASLD among cases and controls was 91 (47.9%) and 64 (33.7%), respectively. T2DM patients are 1.81 times more likely to have MASLD than those without type II DM (OR: 1.81, CI: 1.19-2.74) (Table 2).

**Table 2:** Comparison of metabolic dysfunction-associated liver disease between case and control groups. MASLD: Metabolic dysfunction-associated liver disease.

		Study Groups		Total	P-value	OR
		Case	Control			
MASLD	Yes	91 (47.9%)	64 (33.7%)	155 (40.88%)	0.005	1.810 (1.19-2.74)
	No	99 (52.1%)	126 (66.3%)	225 (59.2%)		

Stratification of patients according to age groups demonstrated that in participants with age less than 50 years, there was no statistically significant difference in terms of MASLD with an OR of 1.36 (0.69-2.69) and p-value of 0.372. In contrast, in the >50(years) age group, MASLD was noted to be 62 (51.2%) among the cases group compared to 39 (33.1%) in the control group, and the difference was statistically significant with an OR of 2.13 (1.26-3.59) (Table 3).

**Table 3:** Comparison of MASLD between case and control groups stratified by age. MASLD: Metabolic dysfunction-associated liver disease.

Age (years)	MASLD	Study Groups		Total	P-value	OR
		Case	Control			
≤50	Yes	29 (42.0%)	25 (34.7%)	54 (38.3%)	0.372	1.36 (0.69-2.69)
	No	40 (58.0%)	47 (65.3%)	87 (61.7%)		
>50	Yes	62 (51.2%)	39 (33.1%)	101 (42.3%)	0.004	2.13 (1.26-3.59)
	No	59 (48.8%)	79 (66.9%)	138 (57.7%)		

The gender-specific stratified analysis revealed that in the male cohort, MASLD was identified in 47 (45.6%) participants from the cases group, in comparison to only 30 (27.8%) in the control group, resulting in an OR of 2.18 (1.23-3.87). Conversely, within the female stratum, the difference in MASLD prevalence between cases and controls was not statistically significant, with a p-value of 0.235 and an OR of 1.44 (0.78-2.65) (Table 4).

**Table 4:** Comparison of MASLD between case and control groups stratified by gender. MASLD: Metabolic dysfunction-associated liver disease.

Gender	MASLD	Study Groups		Total	P-value	OR
		Case	Control			
Male	Yes	47 (45.6%)	30 (27.8%)	77 (36.5%)	0.007	2.18 (1.23-3.87)
	No	56 (54.4%)	78 (72.2%)	134 (63.5%)		
Female	Yes	44 (50.6%)	34 (41.5%)	78 (46.2%)	0.235	1.44 (0.78-2.65)
	No	43 (49.4%)	48 (58.5%)	91 (53.8%)		

According to the BMI, study individuals were divided into two groups: BMI ≤25 kg/m2 and BMI >25kg/m². The results showed that in patients having BMI ≤25 kg/m2 among the cases group, the MASLD was found in 60 (48.0%) patients, while among the control group, it was noted in 43 (35%) patients with OR of 1.72 (1.03-2.86). Similarly, in patients having BMI >25 kg/m2, the MASLD was noted in 32 (49.23%) patients in the cases group compared to 20 (29.85%) in the control group, with a statistically significant OR of 2.28 (1.12-4.66) (Table 5).

**Table 5:** Comparison of MASLD between case and control groups stratified by BMI. MASLD: Metabolic dysfunction-associated liver disease.

BMI	MASLD	Study Groups		Total	P-value	OR
		Case	Control			
≤25	Yes	60 (48.0%)	43 (35.0%)	103 (41.5%)	0.037	1.72 (1.03-2.86)
	No	65 (52.0%)	80 (65.0%)	145 (58.5%)		
>25	Yes	32 (49.23%)	20 (29.85%)	52 (39.4%)	0.024	2.28 (1.12-4.66)
	No	33 (50.77%)	47 (70.15%)	80 (60.6%)		

## Discussion

Fatty liver or hepatic steatosis is characterized by diffuse fat accumulation in hepatocytes. Fatty liver occurring in individuals without a history of significant alcohol intake is termed MASLD. The natural course of MASLD includes steatosis, steatohepatitis, cirrhosis, and, in some instances, hepatocellular cancer. Obesity, T2DM, and hyperlipidemia are all strongly linked to MASLD [7, 21]. In this study, the MASLD was observed in 155 (40.79%) patients. Among the cases group, the MASLD was noted in 91 (47.9%) patients, while among the control group, the MASLD was noted in 64 (33.7%) patients. Statistically, there are 1.810 odds of having MASLD among cases compared to controls, i.e., OR of 1.810 (1.19-2.74). Some of the studies are discussed below, showing their results in favor of our study, as patients with T2DM or pre-diabetes had progressive fibrosis when their serial biopsies were studied. It has also been suggested that the advanced forms of MASLD, such as MASH, advanced fibrosis, cirrhosis, and hepatocellular carcinoma, occur more commonly in these patients [5, 22]. "Yamada T et al. demonstrated in their study that fatty liver constitutes an independent risk factor for impaired fasting glucose and T2DM, exerting a more significant impact on Japanese individuals with a lower BMI undergoing health check-ups [23]. The presence of both MASLD and T2DM increases the likelihood of developing complications of T2DM (including both macro and microvascular complications). It augments the risk of more severe MASLD, including cirrhosis, hepatocellular carcinoma, and death [5]. In cases with T2DM, the frequency of MASLD was 28.3%, while 17.5% in controls without T2DM [6]. The difference was significant (p<0.001). In their study, Dharmalingam M and Yamasandhi PG concluded that T2DM and MASLD have a common association. The increasing prevalence makes it a public health problem [24]. They each have an impact on the other's trajectory. Because of its tight relationship with insulin resistance, steatosis necessitates immediate clinical assessment for signs of metabolic syndrome, insulin resistance, and T2DM. Effective management of T2DM is essential to reduce insulin resistance, which can show amelioration in the MASLD.

Luxmi S et al. conducted a study on the association of MASLD with T2DM [25]. The authors found that out of 120 patients with T2DM, 73 (60.8%) had fatty liver as detected by ultrasonography. There was an increase in BMI and levels of HBA1c, ALT, AST, alkaline phosphatase, Gamma-glutamyl transferase, total cholesterol, triglycerides, and LDL, along with a decrease in HDL, observed in the fatty liver group compared to the non-fatty liver group. Evidence suggests that patients with MASLD and T2DM are at a particularly increased risk of progressive stages of the disease and are at a higher risk of developing cirrhosis compared to MASLD patients without T2DM [26, 27]. Studies demonstrated that the prevalence of MASLD varies widely depending on the population studied and the methodology applied. Studies have shown that MASLD may be present in up to 70% of patients with T2DM [28, 29]. The prevalence of obesity, hypertension, and dyslipidemia was significantly higher in subjects with MASLD. However, our study has certain limitations. Firstly, the current study was conducted within the South Asian population, so the results could not be generalized and further investigations should be carried out to establish generalizability. Secondly, our study is confined to a single-center case-control design, underscoring the need for large-scale, multi-center studies to fortify the robustness of current findings. Lastly, we relied on abdominal ultrasound for MASLD diagnosis, which could introduce limitations related to operator skill and procedural variability. In future research, we recommend incorporating advanced imaging techniques such as elastography and other noninvasive markers of fibrosis to comprehensively assess and evaluate the precise relationship between MASLD and T2DM.

## Conclusions

In conclusion, MASLD is significantly associated with T2DM, regardless of gender and BMI of patients. We recommend screening T2DM patients for the presence of MASLD at regular intervals in order to prevent hazardous consequences of MASLD in adult populations, particularly those with features of metabolic syndrome. Further larger-scale studies investigating the impact of T2DM on MASLD are required to reduce morbidity and decrease disease burden, especially in prevalent areas.

## References

[1] Alavi N, Amin S, Mumtaz M (2016). Non-alcoholic fatty liver disease (NAFLD): frequency in diabetes mellitus (type II) patients and non diabetic group at Shalamar Medical and Dental College, Lahore. Professional Med J.

[2] Byrne CD, Targher G (2015). NAFLD: a multisystem disease. J Hepatol.

[3] Rinella ME (2015). Nonalcoholic fatty liver disease: a systematic review. JAMA.

[4] Patel H, Verma YN (2018). Prevalence of non-alcoholic fatty liver disease in type-2 diabetes mellitus patients. Int J Res Med Sci.

[5] Barba C, Cavalli ST, Cutter J (2004). Appropriate body-mass index for Asian populations and its implications for policy and intervention strategies. Lancet.

[6] Schellenberg ES, Dryden DM, Vandermeer B, Ha C, Korownyk C (2013). Lifestyle interventions for patients with and at risk for type 2 diabetes: a systematic review and meta-analysis. Ann Intern Med.

[7] Younossi Z, Anstee QM, Marietti M (2018). Global burden of NAFLD and NASH: trends, predictions, risk factors and prevention. Nat Rev Gastroenterol Hepatol.

[8] Younossi ZM (2019). Non-alcoholic fatty liver disease - A global public health perspective. J Hepatol.

[9] Kabbany MN, Conjeevaram Selvakumar PK, Watt K (2017). Prevalence of nonalcoholic steatohepatitis-associated cirrhosis in the United States: an analysis of National Health and Nutrition Examination Survey Data. Am J Gastroenterol.

[10] Ludwig J, Sanbonmatsu L, Gennetian L (2011). Neighborhoods, obesity, and diabetes--a randomized social experiment. N Engl J Med.

[11] Shastri M, Rathod VM, Raval DM, Khan S, Mallik S, Solanki DL (2023). Liver involvement in individuals with obesity: a cross-sectional study from Western India. J Assoc Physicians India.

[12] Lu FB, Hu ED, Xu LM (2018). The relationship between obesity and the severity of non-alcoholic fatty liver disease: systematic review and meta-analysis. Expert Rev Gastroenterol Hepatol.

[13] Goh GB, Pagadala MR, Dasarathy J (2015). Clinical spectrum of non-alcoholic fatty liver disease in diabetic and non-diabetic patients. BBA Clin.

[14] Cusi K (2012). Role of obesity and lipotoxicity in the development of nonalcoholic steatohepatitis: pathophysiology and clinical implications. Gastroenterology.

[15] Gaggini M, Morelli M, Buzzigoli E, DeFronzo RA, Bugianesi E, Gastaldelli A (2013). Non-alcoholic fatty liver disease (NAFLD) and its connection with insulin resistance, dyslipidemia, atherosclerosis and coronary heart disease. Nutrients.

[16] Bril F, Cusi K (2017). Management of nonalcoholic fatty liver disease in patients with type 2 diabetes: a call to action. Diabetes Care.

[17] Singh SP, Singh A, Pati GK (2014). A study of prevalence of diabetes and prediabetes in patients of non-alcoholic fatty liver disease and the impact of diabetes on liver histology in coastal eastern India. J Diabetes Mellit.

[18] Vijan S (2010). In the clinic. Type 2 diabetes. Ann Intern Med.

[19] Hazlehurst JM, Woods C, Marjot T, Cobbold JF, Tomlinson JW (2016). Non-alcoholic fatty liver disease and diabetes. Metabolism.

[20] Liu M, Wang J, Zeng J, Cao X, He Y (2017). Association of NAFLD with diabetes and the impact of BMI changes: a 5-year cohort study based on 18,507 elderly. J Clin Endocrinol Metab.

[21] Mulhall BP, Ong JP, Younossi ZM (2002). Non-alcoholic fatty liver disease: an overview. J Gastroenterol Hepatol.

[22] Anstee QM, McPherson S, Day CP (2011). How big a problem is non-alcoholic fatty liver disease?. BMJ.

[23] Yamada T, Fukatsu M, Suzuki S, Wada T, Yoshida T, Joh T (2010). Fatty liver predicts impaired fasting glucose and type 2 diabetes mellitus in Japanese undergoing a health checkup. J Gastroenterol Hepatol.

[24] Dharmalingam M, Yamasandhi PG (2018). Nonalcoholic fatty liver disease and type 2 diabetes mellitus. Indian J Endocrinol Metab.

[25] Luxmi S, Sattar RA, Ara J (2008). Association of non-alcoholic fatty liver with type 2 diabetes mellitus. JLUMHS.

[26] Firneisz G (2014). Non-alcoholic fatty liver disease and type 2 diabetes mellitus: the liver disease of our age?. World J Gastroenterol.

[27] Angulo P, Keach JC, Batts KP, Lindor KD (1999). Independent predictors of liver fibrosis in patients with nonalcoholic steatohepatitis. Hepatology.

[28] Younossi ZM, Gramlich T, Matteoni CA, Boparai N, McCullough AJ (2004). Nonalcoholic fatty liver disease in patients with type 2 diabetes. Clin Gastroenterol Hepatol.

[29] Williamson RM, Price JF, Glancy S (2011). Prevalence of and risk factors for hepatic steatosis and nonalcoholic Fatty liver disease in people with type 2 diabetes: the Edinburgh Type 2 Diabetes Study. Diabetes Care.

